# Effects of Seaweed Extracts on the Growth, Physiological Activity, Cane Yield and Sucrose Content of Sugarcane in China

**DOI:** 10.3389/fpls.2021.659130

**Published:** 2021-05-26

**Authors:** Diwen Chen, Wenling Zhou, Jin Yang, Junhua Ao, Ying Huang, Dachun Shen, Yong Jiang, Zhenrui Huang, Hong Shen

**Affiliations:** ^1^College of Natural Resources and Environment, South China Agricultural University, Guangzhou, China; ^2^Guangdong Provincial Key Laboratory of Eco-Circular Agriculture, Guangzhou, China; ^3^Institute of Bioengineering, Guangdong Academy of Sciences, Guangzhou, China; ^4^Crops Research Institute, Guangdong Academy of Agricultural Sciences, Guangzhou, China; ^5^Guangdong Provincial Key Laboratory of Crop Genetics and Improvement, Guangzhou, China

**Keywords:** seaweed extracts, foliar application, sugarcane (*Saccharum officinarum* L.), photosynthesis, sucrose

## Abstract

Seaweed extracts (SEs) have been widely used as biostimulants in crop management due to their growth-promoting and stress-resistant effects. To date, there are few reports of the effect of SEs on sucrose content and cane yield. Here, we conducted field experiments for three consecutive growth seasons (2017∼2019) in two areas (Suixi and Wengyuan) of China, to investigate the yield and sugar content of sugarcane in response to SE treatment at different growth stages. The results showed that spraying SEs once at seedling (S), early elongation (E), and early mature (M) stages, respectively, once at S and E stages, respectively, or once at the S stage increased the cane yield by 9.23, 9.01, and 3.33%, respectively, implying that SEs application at the early elongation stage played a vital role in promoting sugarcane growth. Photosynthetic parameters and nutrient efficiency analysis showed that spraying SEs at S and E stages enhanced the net photosynthetic rate, transpiration rate, and water use efficiency, and increased N, P, or K utilization efficiency, compared with those of the control. Notably, cane yield increasing rate of SEs in 2017 and 2018 were higher than those in 2019 in Wengyuan but lower than those in 2019 in Suixi. Interestingly, the total rainfall and monthly average rainfall in 2017 and 2018 were lower than those in 2019 in Wengyuan but higher than those in 2019 in Suixi. The results suggested that the yield increasing rate of SEs on sugarcane was better in less rainfall years. The sucrose content of sugarcane showed no difference between spraying SEs at the M stage alone or at the three growth stages but was higher than those of SE treatments at S and/or E stages. Enzyme activity analysis showed that spraying SEs at the M stage increased the activity of sucrose phosphate synthase activity by 9.14% in leaves and 15.16% in stems, and decreased soluble acid invertase activity in stems by 16.52%, which contributed to the sucrose increase of 5.00%. The above results suggested that SEs could increase cane yield and promote sucrose accumulation in sugarcane. The yield increasing effect was more obvious under conditions of drought stress.

## Introduction

Sugarcane is the most important sugar crop and is also an important renewable energy crop (Commodity Bureau, 2015). China is the third largest sugarcane producer in the world after Brazil and India (Faostat, 2016). In recent years, periods of drought have become more frequent and serious due to global climate change (Hoover et al., 2017). Some climate models predict that the occurrence frequency of drought and extreme drought in subtropical and tropical regions will increase in the future, and the impact scope will be larger (Burke et al., 2006). Drought directly causes serious damage to various crops, such as sugarcane. Drought could lead to the reduction of sugarcane yield and have a serious impact on sugarcane agricultural production and the sugar industry (Vasantha et al., 2005). The arid slope areas of Guangxi, Guangdong, and Yunnan are the main sugarcane-growing areas in China. The water source of most sugarcane fields basically depends on rainfall, and there were almost no irrigation measures. Unfortunately, the uneven rainfall in these areas was prone to seasonal drought, which seriously affects the normal growth of sugarcane. How to effectively improve the drought resistance of sugarcane and ensure the yield of sugarcane and sugar has become an important topic in the field of sugarcane research (Kumar et al., 2014; Liu et al., 2016; Pereira et al., 2019; Singh et al., 2019). Thus far, there have been some studies to achieve high-efficiency irrigation management of sugarcane by changing the field application measures (Singh A.K. et al., 2018). Others use soil water retaining agents to improve the soil water holding capacity, promote root water absorption, and improve sugarcane drought resistance to ensure sugarcane yield and sugar content (Marcos et al., 2018; Singh et al., 2018a; Silveira et al., 2019). Nowadays, more and more scholars are interested in improving the stress ability of sugarcane under biological and abiotic stress, by applying exogenous growth stimulating substances (Pereira et al., 2019; Watanabe et al., 2019).

Seaweed extracts (SEs) are a kind of biostimulant extracted from seaweed (especially brown algae) that can promote crop growth, improve crop quality, and enhance crop stress resistance. SE mainly contain natural hormones, such as auxin, cytokinin, gibberellin, abscisic acid, and other active substances such as seaweed polysaccharide, sugar alcohol, betaine, and phenolic compounds (Crouch and van Staden, 1993; Jardin, 2012; Battacharyya et al., 2015), which have been used in agriculture for many years (Friedlander and Ben-Amotz, 1990; Mukherjee and Patel, 2020). The studies have shown that SEs were beneficial to soil improvement and crop growth. The colony counts in the soil and metabolic activities of soil microbes were found to increase following SEs applications, which contributed to increase plant root and shoot growth (Alam et al., 2013). SEs increased the absorption of soil nutrients by plants, stimulated the growth of crops, increased yield (Renaut et al., 2019; Boukhari et al., 2020), and enhanced plant resistance to biotic (Machado et al., 2014; Ben Salah et al., 2018) and abiotic stress (Bradáčová et al., 2016; Cabo et al., 2019; Khompatara et al., 2019). For example, SEs sprayed on onion grown under water stress significantly increased N, P, and K uptake by 116, 113, and 93% compared to the unsprayed plants (Almaroai and Eissa, 2020). Another study found that SEs increased chlorophyll content by increasing the biogenesis of chloroplasts and reducing chlorophyll degradation, which was due to the up-regulated genes associated with photosynthesis, cell metabolism, stress response and S and N metabolism in *Brassica napus* L. (Jannin et al., 2013). Researchers postulated that the stimulatory effect of seaweed extracts on plant growth was due to the complex of active substance, which act directly or by influencing gene regulation in the plant (Arioli et al., 2015). There was a significantly higher expression levels of the PinII and ETR-1 marker genes with SEs application than controls. This was coupled with a marked increase in gene transcripts involved in auxin (IAA), gibberellin (Ga2Ox) and cytokinin (IPT) biosynthesis, which provides possible evidence for induced growth in plants treated with SEs (Ali et al., 2019).

Seaweed extracts have been shown to be effective in improving stress resistance in many other crops, such as spinach (Xu and Leskovar, 2015), maize (Trivedi et al., 2018a,b), sweet orange (Spann and Little, 2011), zucchini squash (Rouphael et al., 2016), and cucumber (Spann and Little, 2011). There are almost no reports on the application of SEs in sugarcane, especially in arid areas without irrigation. Indian researchers have conducted field experiments in western and southern India, which showed that the application of SEs could improve the yield and sugar content of sugarcane (Deshmaukh and Phonde, 2013; Karthikeyan and Shanmugam, 2017). In addition, other reports in India showed that the application of SEs could reduce fertilizer input and increase sugar yield (Deshmaukh and Phonde, 2013; Karthikeyan and Shanmugam, 2017). Meanwhile, it is believed that the application of SEs in sugarcane could reduce carbon dioxide emission and encourage the use of biostimulants, such as SE, under the background of adverse effects of global climate change (Singh et al., 2018b). However, the soil, climate, and cultivation measures between China and India are different. It is necessary to carry out tests to investigate the application effects of SEs on sugarcane in China. Specifically, in conditions without irrigation, the effects of SEs on sugarcane growth, yield, and sucrose content are not clear. We, therefore, conducted a series of field experiments for three consecutive years (1-year planting and 2-year ratoons) in the main sugarcane producing areas of China to investigate the effects of SEs on sugarcane growth, yield, and sugar content in terms of yield components, photosynthetic parameters, nutrient utilization rate, sucrose content, and sugar-related enzyme activities.

## Materials and Methods

### Trial Sites and Weather Data

**FIGURE 1:**
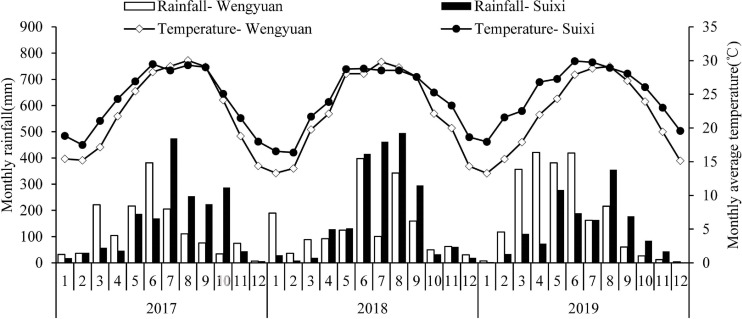
Monthly total rainfall and average temperature during experimental year (2017–2019) in two sites. Numbers in the X axis represent the months in 3 years.

Wengyuan (113.94E, 24.27N) and Suixi counties (110.28E, 21.35N), which both have subtropical climates, were selected as the trial sites. Figure 1 shows the monthly precipitation and monthly average temperature of the two sites from 2017 to 2019. In winter, the temperature in Wengyuan was 3–6°C lower than that of Suixi. The 3-year average temperature in Wengyuan was 22.3°C and that in Suixi was 24.4°C. The rainfall in Wengyuan from 2017 to 2019 was 1,498, 1,674, and 2,183 mm, respectively, and the rainfall in Suixi from 2017 to 2019 was 1,805, 2,098, and 1,514 mm, respectively. The soil in the sugarcane field was latosol at Suixi and red soil at Wengyuan. Soil properties are listed in Table 1.

**TABLE 1:** Physical and chemical properties of experimental soils.

Trial site	pH	Organic matter (g⋅kg^–1^)	Total-N (g⋅kg^–1^)	Available P (mg⋅kg^–1^)	Available K (mg⋅kg^–1^)
Suixi	4.80	14.97	0.92	65.35	140.13
Wengyuan	4.73	20.83	1.17	21.93	66.83

### Plant Materials and SE

The sugarcane variety used in Suixi was “ROC22” and in Wengyuan, it was “Yuetang60.” These two varieties were the main local cultivated varieties.

The raw material of SEs was obtained from cultured kelp in the coastal waters of southeast China. The SEs was a kind of liquid product obtained by complex enzymatic hydrolysis. The content of the main nutrients and active substances in the SE were pH 6.85, EC value 14.35 mS/cm, N 0.56 g/L, P_2_O_5_ 0.28 g/L, K_2_O 12.06 g/L, Ca 3.32 g/L, Mg 2.65 g/L, S 1.68 g/L, organic matter 25.10 g/L, alginic acid 20.16 g/L, seaweed polyphenol 205.56 mg/L, effective seaweed oligosaccharide 4.00 g/L, total sugar 13.00 g/L, mannitol 12.10 g/L, and free amino acid 5.00 g/L.

### Treatments and Cultural Practices

A total of five treatments were set up in the field experiments, and each treatment was repeated four times. Treatments were randomly distributed including: (1) spraying water without SE as a control (CK); (2) spraying SEs once at the seedling stage (SE1); (3) spraying SEs both at seedling and early elongation stages (SE2); (4) spraying SEs at seedling, early elongation, and early mature stages (SE3); and (5) spraying SEs once at the early mature stage (SE4). The purpose of SE5 was only to study the effect of SE on the sugar content of sugarcane. The application amount of SE remained constant at 3 L/ha, which was diluted 100 times with clear water and sprayed by an unmanned aerial vehicle. The application time at the seedling, early elongation, and early mature stages were in late March, mid-June, and early November every year, respectively. There was a slight difference in dates between different years (within 10 days).

Both sites were newly planted in 2017 and ratoons in 2018–2019. The former crops planted at these sites were sugarcane. The planting time of Suixi and Wengyuan was December 27, 2016 and December 10, 2016, respectively. Plot sizes were 168 m^2^ (7 rows × 20 m) and 126 m^2^ (7 rows × 15 m) at Suixi and Wengyuan, respectively. Both sites had a row-spacing of 1.2 m. All experiments had 3–5 guard rows to minimize cross influence. Total fertilization amounts were 483 kg N, 240 kg P_2_O_5_, and 450 kg K_2_O, and the fertilization amount in ratoon (2018/2019) was 432 kg N, 225 kg P_2_O_5_, and 405 kg K_2_O. Cane in all of the experiments was planted and cultivated following local cultivation practices and was harvested after approximately 12 months of growth.

### Measurements

#### Cane Yield and Its Components

On the 15th day after the second spraying treatment in 2017–2018, plant height was measured with a special ruler for measuring the plant height of sugarcane, and 30 plants were randomly measured in each plot to take the average of a sample. Every year (2017–2019) from December 15–20, the diameter of the central stem of sugarcane was measured with a vernier caliper, and 30 plants were randomly measured in each plot to take the average of a sample. The millable cane numbers in the area of 36 m^2^ (3 rows in the middle × 10 m length) were counted in each plot and converted into the millable cane number per hectare. The cane yield (fresh cane weight) was converted into yield per hectare by weighing an area of 36 m^2^ in each plot.

#### SPAD (Soil and Plant Analyzer Development) Value and Photosynthetic Parameters of Leaves

In 2017 and 2018, SPAD value measurements of sugarcane leaves were taken 3 times at 2 weeks after each SE application. A chlorophyll meter (SPAD-502) was used to measure the middle part of the fully expanded leaves at the top of the sugarcane plant, and the average value of 10 plants was taken as a measured value.

In sunny weather, from 9:00 a.m. to 11:00 a.m., using a LI-6400 portable photosynthetic system, the photosynthetic parameters of the middle part of the leaves of sugarcane with red and blue light sources were measured, and 10 plants were measured repeatedly to obtain the average value. The following measurements were recorded: CO_2_ concentration 390.5 μmol/mol, light intensity 800 μ mol/(m^2^s), net photosynthetic rate (*Pn*), and transpiration rate (*Tr*). The ratio of *Pn*/*Tr* was calculated as instant water use efficiency (WUEI).

#### Nutrient Utilization Efficiency (NUE) and Partial Factor Productivity (PFP)

When sugarcane was harvested, 10 sugarcane plants were randomly selected from each plot, and the sugarcane stems and leaves were collected, and fresh weight were weighed, then killed at 105 °C for 30 min and dried to constant weight at 70°C, and dry weight were weighed, and water contents of stems and leaves were calculated. The drying sample was crushed through a 0.15-mm sieve, treated with H_2_SO_4_-H_2_O_2_ followed by wet digestion (Bao, 2000), and the nutrient content of nitrogen (N) and phosphorus (P) contents were analyzed by an Automatic Flow Injection Analyzer (Proxima, Alliance, France), and potassium (K) content was measured with a Flame Emission Spectrophotometer (M425, Sherwood, United Kingdom). The Nutrient content per plant was the sum of the nutrient content of stem and leaf. The nutrient utilization efficiency (NUE) of N, P, and K were calculated as follows:

NUE*_*N/P/K*_* = Nutrient content per plant (*N/P/K*) × millable cane number per hectare/the amount of *N/P/K* fertilizer applied (F*_*N/P/K*_*) per hectare

PFP*_*N/P/K*_* = Cane yield/F*_*N/P/K*_*

#### Contents of Sucrose and Reducing Sugar in Sugarcane, and Theoretical Sugar Yield

The sugar parameters of cane were sampled and tested at each harvest time in 2017–2019. The content of sucrose, glucose, and fructose were determined by HPLC. A total of 10 canes were randomly selected from each plot, and the tenth node (counting from bottom to top) was peeled and cut into small pieces, which were ground into a uniform powder with liquid nitrogen. The powder (2.5 g) was weighed and placed in a 50 mL centrifuge tube, and 10 mL ethanol with a volume fraction of 80% was added. For extraction, the samples were incubated in an 80°C water bath for 30 min, shaken once every 5 min, and centrifuged at 12,000 r⋅min^–1^ for 15 min to collect the supernatant. The extraction was repeated twice with 80% ethanol, and the supernatants of the three extractions were combined in a 50 mL centrifuge tube, which were soaked in a water bath at 90°C for about 3 h, volatilized to about 2 mL, and the supernatant was fixed to 10 mL. The supernatant was filtered with a 0.22 μm microporous membrane to remove impurities and obtain the sugar extract. Chromatographic conditions were as follows: YMC-Pack NH_2_ carbohydrate column (250 mm × 4.6 mm, 5 μm), column temperature 40°C, flow rate 1 mL⋅min^–1^, injection volume 20 μL, and time 20 min. According to the peak area and concentration of the standard sample, the sugar content in the sample was calculated by using the formula: standard sample peak area/standard sample concentration = sample peak area/sample concentration. The content of reducing sugar was the sum of the glucose and fructose content. Theoretical sugar yield was calculated by cane yield per unit area multiplied by sucrose content. Sucrose content, reducing sugar content, and theoretical sugar yield were all based on fresh weight of cane.

#### SAI and SPS Enzyme Activities

In 2018, 10–12 days after spraying SEs at the mature stage, the leaves (completely unfolded at the top of sugarcane) and stems (peeled from the tenth node, counting from bottom to top, and cut into small pieces) were sampled. After picking, they were put into liquid nitrogen until analysis and detection in the laboratory. The sample powder (2 g) was ground with liquid nitrogen, weighed, and put into a 10 mL centrifuge tube, and 8 mL of enzyme extract (50 mM Hepes (pH 7.5), 12 mM MgCl, 1 mM EDTA, 1 mM EGTA, 10 mM DTT, 2 mM benzamidine, 2 mM N-aminocapronate, and 10 mM diethyldithiocarbamate) was added and extracted by shaking on ice for 30 min. Samples were centrifuged at 4°C at 15,000 rpm for 10 min. The supernatant (4 mL) was placed into a 2 mL centrifuge tube. The extraction and enzyme activity of SAI and SPS were determined according to the methods of Zhu et al. (1997) and Gutiérrez-Miceli et al. (2002).

### Statistical Analyses

We used Microsoft Excel 2013 and SPSS 19.0 to analyze the data. The results were expressed as the mean value and standard error. Analysis of variance and average comparison were based on the least significant difference (LSD) test of 5% probability level in the same place and year.

## Results

### Effects of SEs on Photosynthetic Physiology of Leaves

#### SPAD

**FIGURE 2:**
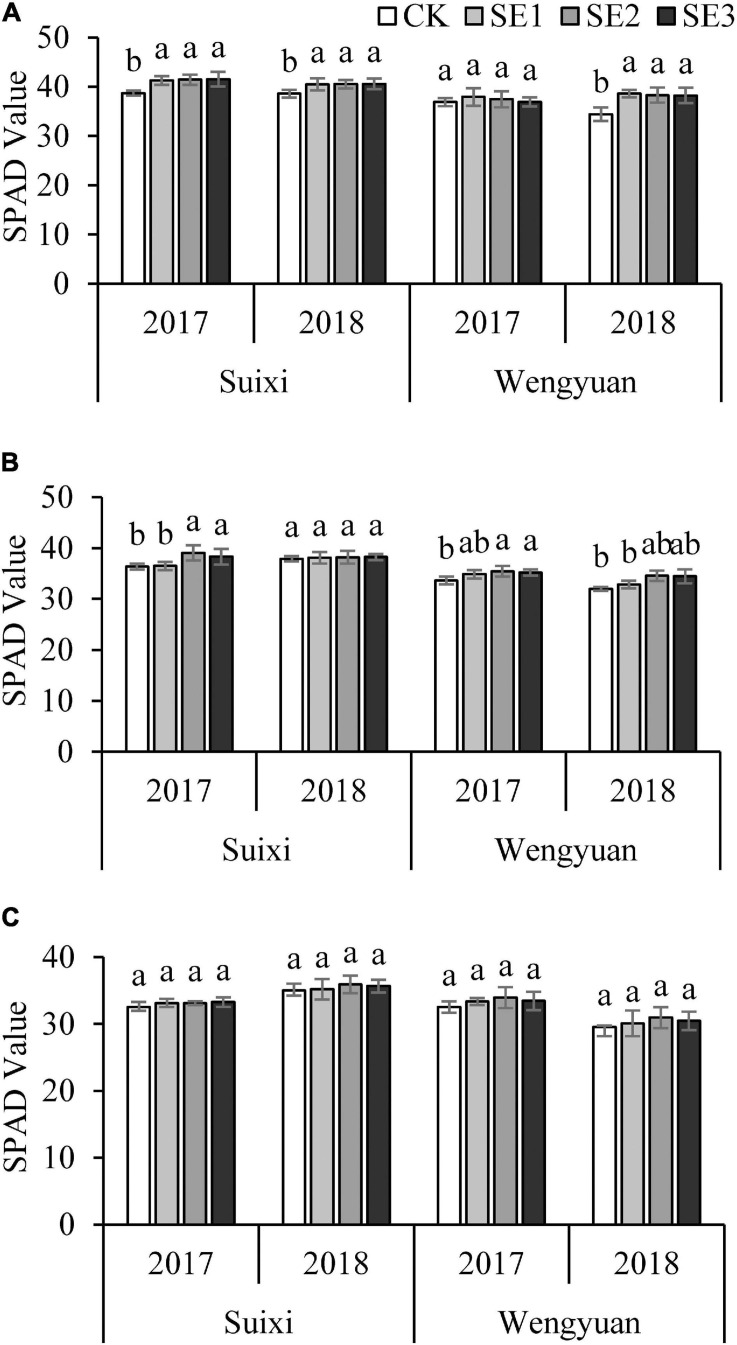
SPAD value of the fully expended top leaf in the seeding **(A)**, Elongation **(B)**, and early maturity **(C)** stages under different SE treatments. ANOVA analysis was conducted among different treatments at the same trial site in the same year and bars with different letters indicate the significance at the 0.05 level.

The SPAD value of leaves increased significantly after spraying SEs at the seedling stage (Figure 2A). Similarly, after the second spraying (SE2/SE3) in the elongation period, SPAD values in plants treated with SEs were significantly higher than that without SE application (Figure 2B), but there was no significant difference between the treatment sprayed with SE only once at the seedling stage and the control treatment. Spraying for the third time at the early mature stage did not affect the SPAD value of leaves (Figure 2C). However, there were differences between different years at the same test site.

#### Pn, Tr, and WUEI

**FIGURE 3:**
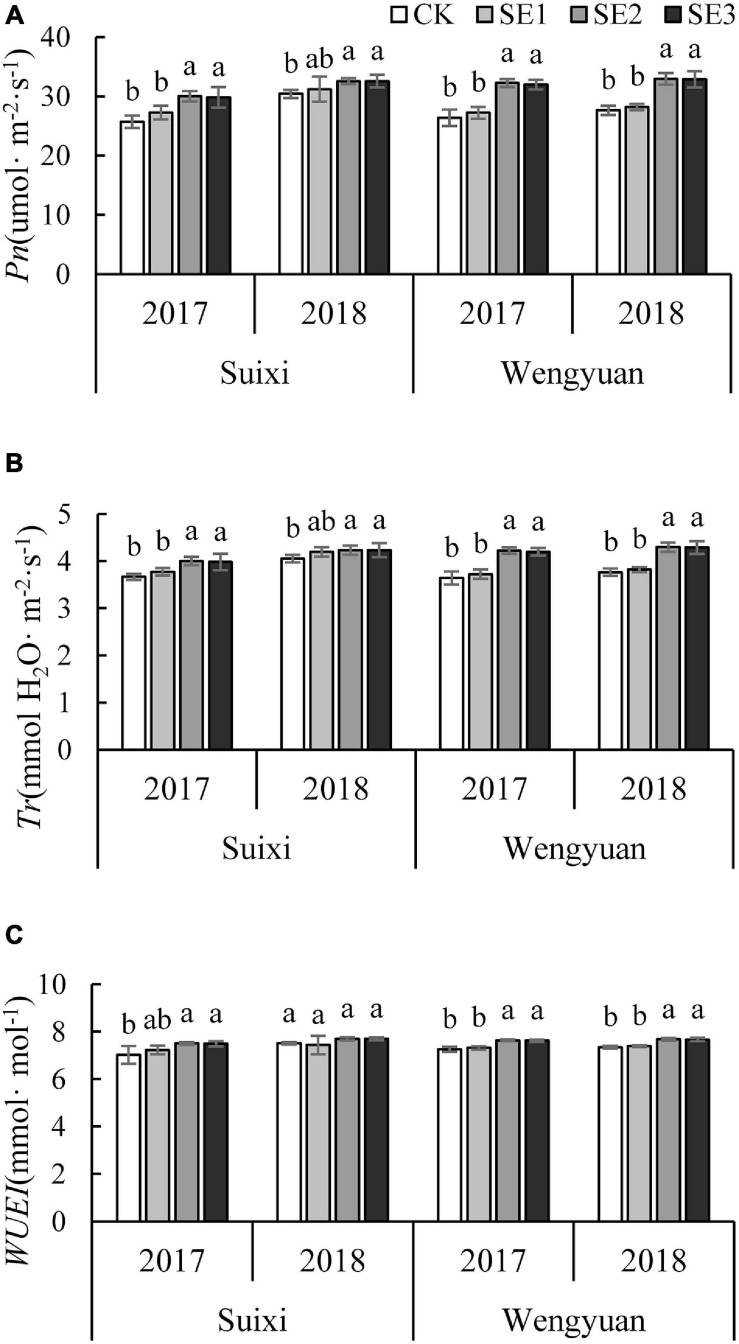
The *Pn*, *Tr*, and *WUEI* of the fully expended top leaf in the Elongation stages under different SE treatments **(A)** *Pn*, **(B)** *Tr*, and **(C)** *WUEI.* ANOVA analysis was conducted among different treatments at the same trial site in the same year and different years and bars with different letters indicate the significance at the 0.05 level.

SE application at the early elongation period (SE2 and SE3) made *Pn* significantly higher than that of the no SE treatment, with an average increase of 14.52%. All four experiments had the same performance (Figure 3A). However, treatment of SE application only once at the seedling stage (SE1) did not have the same effect. Furthermore, the *Pn* of SE2 and SE3 treatments were significantly higher than that of SE1 in three trials (Suixi in 2017, Wengyuan in 2017 and 2018).

The results of *Tr* were similar to *Pn*. The *Tr* of sugarcane sprayed with SEs during the elongation period was significantly higher than that of the control, with an average increase of 10.62%. All experimental results were consistent (Figure 3B). In addition, *Tr* of SE2 and SE3 were significantly higher than that of SE1 in 3 trials (Suixi in 2017, Wengyuan in 2017 and 2018).

The *WUEI* of SE2 and SE3 was significantly higher than that of the control in the elongation period, with an average increase of 4.70% (Figure 3C). Furthermore, WUEI in SE2 and SE3 was significantly higher than that of SE1 in Wengyuan.

### Effects of SEs on Nutrient Utilization Efficiency (NUE) of N, P, and K

The data shown in Table 2 comprise the NUE of N, P, and K of sugarcane, which are the average of six experimental results in two sites for 3 years. The results showed that the NUE of N, P, and K sprayed with SEs were all improved to a certain extent compared with the control. In SE1, SE2, and SE3, the NUE of N increased by 9.88, 17.64, and 18.74%, respectively, of P by 3.26, 13.31, and 14.31%, respectively, and of K by 5.48, 13.70, and 14.49%, respectively. In addition, the N, P, and K PFP of the two treatments (average of SE2 and SE3) were increased by 19.71, 38.42, and 21.06 kg/kg, respectively, compared with the control but the differences were not significant.

**TABLE 2:** N, P, K utilization efficiency and partial productivity of sugarcane under different treatments.

Treatment	Utilization efficiency (%)			Partial productivity (kg/kg)		
	N	P	K	N	P	K
CK	32.87 ± 2.23*ab*	7.96 ± 0.62c	42.75 ± 2.86b	216.08 ± 18.06*a*	421.32 ± 33.32*a*	230.95 ± 19.08*a*
SE1	36.12 ± 4.00*a*b	8.22 ± 0.91*bc*	45.09 ± 4.67*ab*	223.36 ± 20.89a	435.46 ± 37.87a	238.73 ± 22.04a
SE2	38.67 ± 2.43a	9.02 ± 0.87*ab*	48.61 ± 4.09a	235.55 ± 22.08a	459.28 ± 40.85a	251.76 ± 23.35a
SE3	39.03 ± 5.01a	9.1 ± 0.5a	48.95 ± 3.95a	236.01 ± 20.72a	460.2 ± 38.52a	252.25 ± 21.91a

*^*a*^Data presented as mean ± SE, n = 4. Different letters in each column show significant difference at p <0.05, according to LSD.*

### Effects of SEs on Yield Components of Sugarcane

The plant height of sugarcane sprayed with SEs in April was significantly higher than that of CK, and there were significant differences in 4 of 6 experiments (Table 3). After spraying SEs for the second time in the elongation period (SE2/SE3), the plant height of sugarcane in August was significantly higher than that of the control, and this effect was also shown in four experiments. However, spraying SEs for the third time had no significant effect on plant height. According to the results of 3 years of experiments at two sites, compared with the non-SE application, SE3, SE2, and SE1 increased the height of sugarcane by 4.81, 4.66, and 2.04%, respectively. In addition, SE application had no significant effect on the millable cane per unit area (Table 3). Therefore, the promotion effect of SEs on sugarcane growth was mainly reflected in the increase of sugarcane plant height.

**TABLE 3:** Agronomic characters of sugarcane under different treatments.

Site/Year	Treatment	Plant height (cm)			Stalk diameter (mm)	Millable stalks (plant⋅hm^–2^)
		Apr.	Aug.	Dec.	Dec.	Dec.
Suixi/2017	CK	60.97 ± 1.92b^*a*^	212.25 ± 1.79c	285.85 ± 3.63b	32.79 ± 0.64a	54058 ± 1355a
	SE1	67.5 ± 1.39a	217.02 ± 1.01b	290.45 ± 1.1ab	32.61 ± 0.45a	54390 ± 633a
	SE2	68.65 ± 0.88a	236.65 ± 1.77a	298.52 ± 3.74a	33.07 ± 0.33a	54896 ± 599a
	SE3	67.25 ± 2.17a	238.22 ± 3.29a	301.4 ± 1.39a	33.21 ± 0.54a	54087 ± 967a
Suixi/2018	CK	72.67 ± 0.35b	216.1 ± 1.05a	284.05 ± 1.61b	32.11 ± 0.27a	55498 ± 974a
	SE1	77.35 ± 1.6a	220.87 ± 2.01a	289 ± 2.61ab	32.51 ± 0.66a	55229 ± 1280a
	SE2	79.57 ± 1.13a	225.9 ± 4.34a	293.85 ± 2.02a	32.89 ± 0.29a	55641 ± 885a
	SE3	77.17 ± 2.26a	223.85 ± 5.39a	291.52 ± 2.24a	32.93 ± 0.96a	55223 ± 787a
Suixi/2019	CK	70.21 ± 1.02b	206.43 ± 2.47b	268.28 ± 1.24b	31.56 ± 0.20a	52720 ± 736a
	SE1	78.17 ± 4.45a	212.75 ± 1.9a	273.36 ± 2.4ab	31.66 ± 0.28a	52751 ± 545a
	SE2	77.92 ± 1.02a	217.76 ± 1.41a	283 ± 2.45a	32.07 ± 0.17a	52937 ± 614a
	SE3	80.26 ± 4.48a	217.13 ± 1.44a	284.27 ± 1a	32.16 ± 0.49a	52604 ± 732a
Wengyuan/2017	CK	50.47 ± 2.77b	209.77 ± 2.91b	275.95 ± 3.34b	32.07 ± 0.62a	52926 ± 1328a
	SE1	57.97 ± 1.53a	214.55 ± 2.97ab	281.5 ± 3.31b	32.17 ± 0.40a	53116 ± 865a
	SE2	57.9 ± 2.27a	222.5 ± 4.02a	291.7 ± 2.68a	32.56 ± 0.53a	52672 ± 746a
	SE3	57.55 ± 1.28a	224.95 ± 3.27a	292 ± 1.81a	32.68 ± 0.37a	52641 ± 521a
Wengyuan/2018	CK	63.57 ± 1.18b	207.72 ± 4.07b	276.82 ± 3.02b	32.64 ± 0.27a	53519 ± 1417a
	SE1	70.07 ± 0.63a	213.27 ± 1.93ab	285.32 ± 4.91ab	32.93 ± 0.45a	54009 ± 420a
	SE2	69.55 ± 1.21a	218.17 ± 2.77a	291.35 ± 3.41a	33.46 ± 0.16a	53549 ± 1440a
	SE3	69.8 ± 1.58a	222.32 ± 2.55a	292.15 ± 2a	33.42 ± 0.12a	53715 ± 960a
Wengyuan/2019	CK	66.67 ± 2.57a	200.28 ± 2.04b	262.61 ± 2.02b	31.67 ± 0.39a	51326 ± 1107a
	SE1	67.24 ± 0.96a	204.46 ± 1.59ab	267.57 ± 1.37ab	31.66 ± 0.33a	51663 ± 522a
	SE2	68.35 ± 0.84a	209.07 ± 1.42a	272.13 ± 1.1a	31.76 ± 0.36a	51714 ± 622a
	SE3	67.89 ± 1.34a	208.77 ± 1.33a	271.69 ± 1.2a	31.90 ± 0.47a	51781 ± 816a

*^*a*^Data presented as mean ± SE, n = 4. Different letters in each column show significant difference in the same year, the same site at p <0.05, according to LSD.*

### Effects of Different Treatments on Sugarcane Yield

Spraying SEs both at seedling and elongation stages improved sugarcane yield significantly, and the yields of SE2 and SE3 were increased by 9.01 and 9.23%, respectively, compared with those of sugarcane without SE, while the yield of sugarcane sprayed with SEs once at the seedling stage was not significantly different from the control (with a 3.33% yield increase).

The effect of SEs on yield varied with different years and sites. SE treatment (SE3, SE2, and SE1) increased the yield by 6.17, 6.96, and 7.80%, respectively, in Suixi and by 7.59, 9.71, and 4.69%, respectively, in Wengyuan from 2017 to 2019 compared with the control. The 3-year average yield of SE2 and SE3 treatments significantly increased 9.17 and 8.95%, respectively, in Suixi and by 8.83 and 9.53%, respectively, in Wengyuan. However, the yields showed no significant difference between SE2 and SE3 treatments. Similarly, there was no difference in yield between SE4 treatment and CK, which indicated that spraying SEs at the mature stage had no significant effect on yield.

### Sucrose Content, Reducing Sugar Content, and Sugar Yield of Sugarcane in Harvest Period

#### Sucrose Content

Regardless of whether SEs were sprayed in both seedling and elongation stages, the sucrose content of cane with SE application in the mature stage (SE3/SE4) was significantly higher than that of cane without SE application, and the sucrose content of SE3 and SE4 in Suixi in 3 years was 5.71 and 5.49% higher, respectively, than that of the control (Table 8). Furthermore, the increase in Wengyuan was 4.72 and 4.11%, respectively. The average in 3 years at the two sites of SE3 and SE4 treatments significantly increased by 5.21 and 4.79%, respectively (total average 5.00%), compared with those of the control (*P* < 0.05). Notably, SE3 and SE4 treatments were also significantly higher than SE1 and SE2 treatments.

#### Reducing Sugar

Spraying SEs at the mature stage significantly decreased the reducing sugar content of sugarcane by 32.56 and 34.32% (average 33.44%) than that of SE1 and SE2, respectively (Table 4). However, there was no significant difference in reducing sugar content between SE1 and SE2 treatments and those without SE treatment.

**TABLE 4:** Reducing sugar content of sugarcane with different treatments at harvest.

Treatments	Suixi (%)				Wengyuan (%)				Total average (%)
	2017	2018	2019	AVERAGE	2017	2018	2019	Average	
CK	0.47 ± 0.06*a*^a^	0.37 ± 0.1a	0.32 ± 0.01a	0.39 ± 0.05a	0.4 ± 0.05a	0.46 ± 0.06a	0.32 ± 0.01a	0.43 ± 0.05a	0.41 ± 0.05a
SE1	0.43 ± 0.08a	0.37 ± 0.06a	0.3 ± 0.01a	0.37 ± 0.02*ab*	0.41 ± 0.04a	0.43 ± 0.03a	0.3 ± 0.01a	0.43 ± 0.02a	0.4 ± 0.03a
SE2	0.46 ± 0.08a	0.34 ± 0.05a	0.3 ± 0.01a	0.37 ± 0.01b	0.38 ± 0.05a	0.42 ± 0.01a	0.3 ± 0.01a	0.42 ± 0.02a	0.39 ± 0.03a
SE3	0.31 ± 0.07b	0.24 ± 0.05b	0.21 ± 0b	0.25 ± 0.01*c*	0.28 ± 0.03b	0.31 ± 0.01b	0.21 ± 0b	0.3 ± 0.01b	0.28 ± 0.02b
SE4	0.29 ± 0.02b	0.25 ± 0.05b	0.21 ± 0.03b	0.25 ± 0.03*c*	0.28 ± 0.04b	0.28 ± 0.04b	0.21 ± 0.03b	0.29 ± 0.02b	0.27 ± 0.03b

*^*a*^Data presented as mean ± SE, n = 4. Different letters in each column show significant difference in the same year, the same site at p <0.05, according to LSD.*

#### Theoretical Sugar Yield

The 3-year average of sugar content per unit area in Suixi and Wengyuan was significantly higher than that in non-SE treatments, and SE3 was the highest in every year and each place, which was significantly higher than all other treatments. Compared with the control, the sugar content per unit area of SE3 treatment in Suixi and Wengyuan was significantly increased by 15.31 and 16.56%, respectively (*P* < 0.05) and the comprehensive average was increased by 15.92%. In addition, SE1, SE2, and SE4 treatments increased by 5.43, 12.60, and 7.83%, respectively, compared with those of the control (Table 5).

**TABLE 5:** Theoretical sugar yield of sugarcane with different treatments at harvest time.

Treatment	Suixi (t⋅hm^–2^)				Wengyuan (t⋅hm^–2^)				Total average (t hm^–2^)
	
	2017	2018	2019	Average	2017	2018	2019	Average	
CK	17.00 ± 1.01*c*^a^	16.71 ± 0.16c	14.46 ± 0.53d	16.05 ± 0.55*e*	15.15 ± 0.72d	16.48 ± 0.77c	13.94 ± 0.20c	15.19 ± 0.37d	15.62 ± 0.42*e*
SE1	17.15 ± 1.00c	17.65 ± 0.76c	15.41 ± 0.4c	16.74 ± 0.42d	16.09 ± 0.53c	17.73 ± 0.38*bc*	14.80 ± 0.47*bc*	16.21 ± 0.42c	16.47 ± 0.41d
SE2	18.61 ± 0.74b	18.75 ± 0.60a	16.69 ± 0.58a	18.01 ± 0.32b	17.44 ± 0.54b	18.69 ± 0.72a	15.37 ± 0.34a	17.17 ± 0.26b	17.59 ± 0.27b
SE3	19.35 ± 0.66a	18.99 ± 1.02a	17.20 ± 0.56a	18.51 ± 0.51a	17.86 ± 0.43a	19.38 ± 0.42a	15.88 ± 0.5a	17.71 ± 0.21a	18.11 ± 0.33a
SE4	18.18 ± 1.10b	17.80 ± 0.58b	15.70 ± 0.33b	17.23 ± 0.53c	16.90 ± 0.56c	17.52 ± 0.60b	14.98 ± 0.39b	16.47 ± 0.45c	16.85 ± 0.09c

*^*a*^Data presented as mean ± SE, n = 4. Different letters in each column show significant difference in the same year, the same site at p <0.05, according to LSD.*

### Effects of SEs on the Activity of Sucrose Phosphate Synthase (SPS) and Soluble Acid Invertase (SAI)

Compared with the treatment without SE, the activity of SPS in leaves of SE3 and SE4 treatments increased by 9.34 and 8.95% (average 9.14%), respectively, and the SPS enzyme activity in stalks increased significantly by 15.56 and 14.76% (average 15.16%), respectively (*P* < 0.05). However, the activities of SPS in leaves and stems of sugarcane treated with SE1 and SE2 did not change significantly. The SAI enzyme activity in stalks of SE3 and SE4 treatments significantly decreased by 15.20 and 17.84%, respectively (average 16.52%, *p* < 0.05), and was also significantly lower than that of the SE1 treatment (Table 6).

**TABLE 6:** Activities of SPS and SAI in leaves and stems of different treatments at mature stage.

	Treatment	SPS (μmol Suc g^–1^Fw h^–1^)		SAI (μmol Glu g^–1^Fw h^–1^)	
		Suixi	Wengyuan	Suixi	Wengyuan
Leaf	CK	453.65 ± 20.36*b*^a^	462.36 ± 34.68b	870.63 ± 44.44a	972.5 ± 81.75a
	SE1	455.45 ± 20.99b	469.05 ± 20.94b	894.81 ± 25.68a	936.16 ± 85.06a
	SE2	449.75 ± 22.23b	472.31 ± 10.83b	905.42 ± 20.50a	959.75 ± 86.07a
	SE3	489.42 ± 7.96a	512.25 ± 15.79a	888.87 ± 45.17a	946.25 ± 94.7a
	SE4	481.50 ± 13.17a	516.75 ± 13.04a	922.25 ± 51.69a	953.55 ± 66.26
Stalk	CK	228.56 ± 11.89b	255.85 ± 20.01b	359.14 ± 29.94a	332.89 ± 14.47a
	SE1	244.06 ± 26.83b	259.80 ± 13.68b	336.85 ± 20.43a	345.85 ± 13.09a
	SE2	247.30 ± 22.71b	254.60 ± 5.10b	363.31 ± 24.99a	343.33 ± 11.84a
	SE3	274.61 ± 7.46a	283.92 ± 10.67a	289.60 ± 31.12b	296.14 ± 23.90b
	SE4	271.35 ± 16.85a	287.85 ± 9.77a	283.99 ± 8.21b	294.81 ± 5.11b

*^*a*^Data presented as mean ± SE, n = 4. Different letters in each column show significant difference in the same organ, the same site at p <0.05, according to LSD.*

### Correlation Analysis Between Annual Rainfall and Plant Height, Sucrose Content, Cane Yield and Cane Yield Increase

**FIGURE 4:**
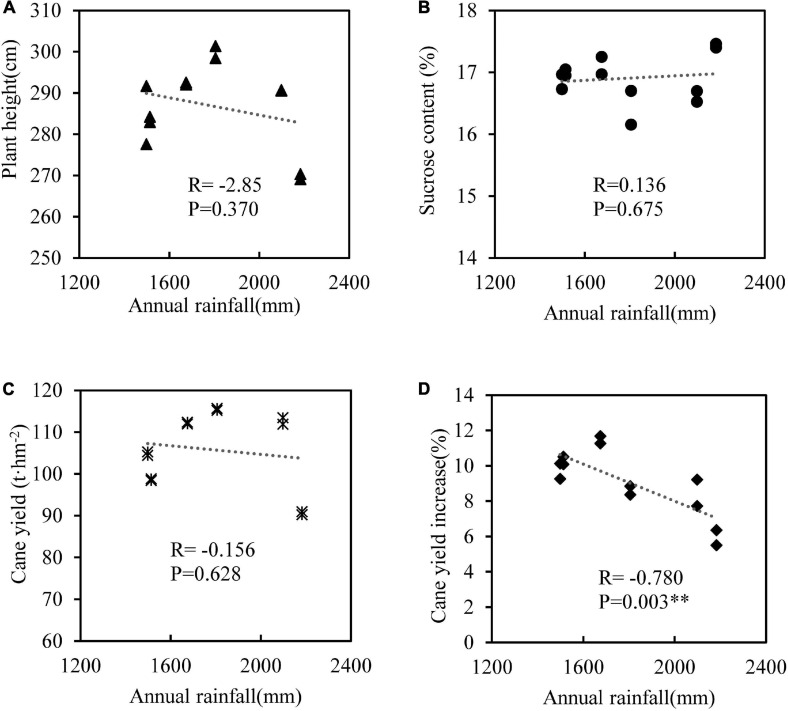
Correlation analysis between annual rainfall and plant height **(A)**, sucrose content **(B)**, cane yield **(C),** and cane yield increase **(D)** with SE2 and SE3 treatments. *n* = 12, ^∗∗^denotes *p* < 0.01.

We used the data of SE2 and SE3 treatments in which SEs had the best effects on sugarcane yield to analyze the correlation of plant height, sucrose content, cane yield and cane yield increase (relative to the treatment without SEs) with annual rainfall. The results showed that plant height, sucrose content and cane yield had no significant correlations with annual rainfall, but the increase of cane yield had a significant negative correlation with rainfall (Figure 4), that means the lower the annual rainfall, the greater the increase in sugarcane yield from SEs application.

## Discussion

### Effects of SEs on Sugarcane Growth and Cane Yield

Biostimulatory activities of SEs were evident throughout the experiments, shown by significant increases in plant height and cane yield. The results of this study are also in agreement with reports on other crops, including strawberry, maize, and tomato (Alam et al., 2013; Ali et al., 2016; Trivedi et al., 2018a,b). The yields of the treatments with SE application (SE1, SE2, and SE3) were higher than those without SE application, with the average increase range of 2.67–9.17% in Suixi and 4.04–9.53% in Wengyuan (Table 7). It has been reported that the application of SE had a better effect on the increase of cane yield. One study showed that SE application both in the soil and on the leaves increased the yield of sugarcane by 14.1% (Deshmaukh and Phonde, 2013), and another experiment showed that spraying SEs on sugarcane three times could achieve a yield increase of 20.47–28.79% (Karthikeyan and Shanmugam, 2017). In terms of yield components, seaweed extract had the greatest effect on plant height, but had no significant effect on stem diameter and millable cane number. The growth promoting properties observed may be a result of the effects of growth regulatory substances present in SE, including low molecular weight biostimulants (seaweed oligosaccharides) that can promote crop growth and high molecular weight biostimulants (algal polysaccharides) that can improve crop stress resistance. These substances induced the biosynthesis of hormones such as phytohormones abscisic acid, cytokinin, and auxin in treated plants (Khan et al., 2009; Aremu et al., 2016; Patel et al., 2018; Ali et al., 2019; Renaut et al., 2019; Mukherjee and Patel, 2020). It has been reported that the yield increasing with SEs applications was associated with improved chlorophyll biosynthesis (higher SPAD index) (Youssef et al., 2018). Our results determined at seedling and elongation stages showed that spraying SEs could significantly increase the SPAD value of sugarcane leaves, which indicated that the application of SEs increased chlorophyll content in leaves (Figure 2), which is also supported by other reports (Lingakumar et al., 2004; Ali et al., 2016, 2019). This might be due to the existence of betaine, amino acids, and other active substances in SEs that inhibit the degradation of chlorophyll (Blunden et al., 1996), Seaweed extracts also contain magnesium, which is necessary for chlorophyll synthesis (Almaroai and Eissa, 2020). Our results showed that spraying SEs had a significant effect on the photosynthetic rate of sugarcane leaves which were consistent with those of SPAD, and these resulted in a stronger ability of plants to maintain a better photosynthetic performance (Santaniello et al., 2017).

**TABLE 7:** Cane yield under different treatments.

Site	Treatment	2017 (t⋅hm^–2^)	2018 (t⋅hm^–2^)	2019 (t⋅hm^–2^)	Average (t⋅hm^–2^)
Suixi	CK	106.30 ± 6.36*b*^a^	103.88 ± 1.68b	89.44 ± 2.25b	99.87 ± 2.98b
	SE1	107.69 ± 3.27b	107.97 ± 3.29b	91.96 ± 1.00b	102.54 ± 1.75b
	SE2	115.19 ± 2.01a	113.45 ± 1.79a	98.46 ± 2.42a	109.03 ± 1.61a
	SE3	115.70 ± 5.38a	111.9 ± 5.35*ab*	98.84 ± 2.63a	108.82 ± 2.98a
	SE4	107.21 ± 7.33b	104.73 ± 1.39b	90.19 ± 3.14b	100.71 ± 3.79b
Wengyuan	CK	95.60 ± 2.65b	100.62 ± 4.99b	85.54 ± 1.88*c*	93.92 ± 1.72b
	SE1	98.84 ± 4.10b	106.86 ± 2.57*ab*	87.44 ± 2.49*bc*	97.71 ± 2.92b
	SE2	104.45 ± 4.28a	111.94 ± 2.83a	90.24 ± 2.36*ab*	102.21 ± 1.24a
	SE3	105.29 ± 3.21a	112.36 ± 3.32a	90.97 ± 1.60a	102.87 ± 0.93a
	SE4	99.07 ± 3.19b	102.82 ± 2.31b	86.54 ± 1.34*c*	96.14 ± 1.40b

*^*a*^Data presented as mean ± SE, n = 4. Different letters in each column show significant difference in the same year, the same site at p<0.05, according to LSD.*

**TABLE 8:** Sucrose content of sugarcane with different treatments at harvest time.

Treatment	Suixi(%)				Wengyuan (%)				Total average (%)
	2017	2018	2019	Average	2017	2018	2019	Average	
CK	15.99 ± 0.08*c*^a^	16.10 ± 0.38b	16.95 ± 0.40b	16.08 ± 0.12b	16.09 ± 0.37b	16.52 ± 0.30b	16.95 ± 0.40b	16.52 ± 0.30b	16.13 ± 0.11b
SE1	15.91 ± 0.55*c*	16.34 ± 0.35b	16.93 ± 0.35b	16.34 ± 0.22b	16.28 ± 0.30b	16.59 ± 0.18b	16.93 ± 0.35b	16.60 ± 0.26b	16.47 ± 0.26b
SE2	16.16 ± 0.77*bc*	16.53 ± 0.32*ab*	17.12 ± 0.56*ab*	16.54 ± 0.28b	16.37 ± 0.34b	16.7 ± 0.64*ab*	17.12 ± 0.56*ab*	16.73 ± 0.42b	16.68 ± 0.35b
SE3	16.73 ± 0.29*ab*	16.97 ± 0.24a	17.69 ± 0.19a	17.03 ± 0.24a	16.96 ± 0.22a	17.25 ± 0.14a	17.69 ± 0.19a	17.30 ± 0.10a	17.13 ± 0.21a
SE4	16.96 ± 0.2a	17.25 ± 0.21a	17.51 ± 0.29a	17.06 ± 0.14a	17.06 ± 0.09a	17.04 ± 0.32a	17.51 ± 0.29a	17.20 ± 0.17a	17.10 ± 0.18a

*^*a*^Data presented as mean ± SE, n = 4. Different letters in each column show significant difference in the same year, the same site at p<0.05, according to LSD.*

Nutrient absorption is an important factor for high yield of crops, and more nutrient absorption leads to higher cane yields (Rhodes et al., 2018). The results showed that the utilization efficiency of nitrogen, phosphorus and potassium in sugarcane sprayed with SEs was significantly higher than that in the treatment without SEs. Previous studies have shown that the application of SEs can promote the growth of crop roots, improve the ability of roots to absorb macroelement nutrients and transfer them to the aboveground (Crouch et al., 1990), which is related to the hormones substances in SE (Finnie and van Staden, 1985; Jeannin et al., 1991), and the hormones substances may promote up-regulated expression of nutrient transport genes, thus improving root uptake and transport of nutrients (Krouk et al., 2010; Rathore et al., 2009). Further studies in the promotion mechanism for SEs regulating plant root growth confirmed that SEs could up-regulated the gene expression and enzyme activity of nitrate reductase at the post-transcriptional level (Zhang et al., 2013).

### Effects of SEs on Sugar Accumulation of Sugarcane

Sucrose content is the most important quality index of sugarcane. All over the world, the sugarcane industry has tried to find ways to improve the sucrose content of sugarcane or accelerate the ripening of sugarcane, among which growth regulator substances are the most frequently used (Rossetto et al., 2003; Li, 2004; Van Heerden et al., 2015; Cunha et al., 2017). In the management of sugarcane, sugar increasers or ripening agents can be applied at the early mature period to advance the harvest period or increase the sucrose content. SEs are a kind of biostimulant that contains a variety of plant hormones, which could regulate the growth and development of plants (Khan et al., 2009; Craigie, 2011; Ali et al., 2016). Therefore, our experiment also set the third application time at the early mature period (from the end of October to November) to evaluate its effects on the sucrose content of sugarcane. Furthermore, we set the treatment of spraying SEs alone in the early mature period (SE4) and compared it with SE3. The results showed that the sucrose content of canes with SE treatment (SE3 and SE4) were significantly increased in comparison with that of non-SE and the SE1 treatment at the early stage of maturity. It was even higher than that of the SE2 treatment in some experiments, and there was no significant difference between SE3 and SE4. These results indicated that the increase of sucrose was mainly due to the SE application at the mature stage, rather than at the seedling and elongation stages.

Sugar conversion and accumulation in sugarcane is regulated by many enzymes, including sucrose synthase (SS), sucrose phosphate synthase (SPS), and invertase (INV) (Sturm, 1999; Winter and Huber, 2000). SPS is a key regulatory enzyme in the distribution of photosynthetic products to sucrose and starch in plants and is positively correlated with sucrose accumulation (Grof et al., 2007). INV is one of the key enzymes controlling sucrose metabolism in plants, which irreversibly catalyzes the conversion of sucrose + H_2_O into fructose + glucose. SAI is a kind of INV, which mainly exists in vacuoles and plays a role in regulating sucrose and hexose levels (Tian et al., 2009), and cane sugar is negatively correlated with SAI activity. In this study, SPS in the direction of sucrose synthesis and SAI in the direction of decomposition and transformation were selected as representatives for analysis. The results showed that spraying SEs at the early stage of maturity significantly increased the activity of SPS enzymes in leaves and stems, which was beneficial to the synthesis and accumulation of sucrose. For stems, SE application significantly reduced the activity of the SAI enzyme, which reduced the decomposition of sucrose in sugarcane stems. Therefore, it could be speculated that SEs regulated related enzymes in sugarcane, promoted the synthesis and accumulation of sucrose, and reduced the transformation to reducing sugar, thus, improving the sucrose content of sugarcane. Reports have shown the elicitation of various plant enzymes and the increasing in the activity of these enzymes by SEs (El Modafar et al., 2012; Ali et al., 2019). The regulation of enzymes activity observed may be as a result of the effects of phytohormones and growth regulatory substances present in the SEs and induced the biosynthesis of hormones by treated plants (Kang et al., 2014; Ramkissoon et al., 2017).

Sucrose accumulation related to biosynthesis of plant hormone signal transduction, which are consistent with the physiological effects elicited by exogenous hormone substances application on sugarcane. While the hormone in signal transduction at the maturation stage are great different from other stage in sugarcane (Cunha et al., 2017). Therefore, the ideal effect can be obtained only when it is applied at mature stage.

### Importance of Applying Seaweed Fertilizer in Rain-Fed Agricultural Areas

Cane yield is closely related to climate factors (Inman-Bamber and Smith, 2005; Liu et al., 2016). In this study, the cane yield of the two experimental sites were different in different years, and the effect of seaweed extract on the yield was also different in different years. In this study, the annual rainfall of the two sites were different (Figure 1 and Table 1). Sugarcane has a great demand for water during the period of rapid growth (May to August every year) in China. The growth of sugarcane is severely inhibited if there was no rainfall and irrigation, resulting in a decline in yield. The uneven seasonal rainfall was also different in the two places. Specifically, the rainfall at Wengyuan in 2017 and 2019 was mainly distributed in March to July and less in the later months, while in June to September 2018, it was less distributed in the early months (Figure 1). In Wengyuan, the highest yield of the 3 years was in 2018 (Table 7), which might be related to the high coincidence between the rainy season and rapid growth of sugarcane in that year. However, the yield in 2019 was lowest, and there were two reasons for this. First, the rainfall decreased significantly after July in that year compared with previous years, which affected sugarcane growth; second, as the second year of ratoon, the emergence of sugarcane generally decreased with the increase of ratoon years, which led to the decrease of millable cane number per unit area (one of the yield components) (Table 3). The rainfall in Suixi from 2017 to 2019 was less in January to May, and more in June to September, especially in 2019. There was no significant difference in sugarcane production between 2017 and 2018 because the rainy season in these 2 years basically coincided with the rapid growth stage of sugarcane. The lowest output in 2019 was due to similar reasons as that of Wengyuan. The effects of SE application on yield also had year-to-year differences. SE treatments had different improvement on sugarcane yield in different years and places, among which Suixi had the largest improvement (>9%) in 2019, which had the least annual total rainfall (1513.72 mm) and monthly average rainfall (126.14 mm). The increase of yield at Wengyuan in 2017 and 2018 was > 10% and < 6% in 2019. Correspondingly, the total rainfall (1498.66 mm/1674.83 mm) and monthly rainfall average (124.89 mm/139.57 mm) in 2017 and 2018 were lower than those in 2019 (rainfall values were not shown in Figure 1). The yield increase rates of SE1, SE2, and SE3 treatments in Suixi were 2.67, 9.17, and 8.95%, respectively, and those of Wengyuan were 4.04, 8.83, and 9.53%, respectively. These results indicated that spraying SEs only once at the seedling stage was not enough to improve yield but spraying SEs two or three times at different growth stages was better.

In addition, the WUEI analysis could also well correspond with this result. The results showed that the WUEI of plants sprayed with SEs in the early elongation stage was significantly higher than that without SEs application, which indicated that SEs could improve the water use efficiency and drought resistance of crops (Neily et al., 2010; Trivedi et al., 2018a,b). Research showed that SEs induced a partial stomatal closure, associated with changes in the expression levels of genes involved in ABA-responsive and antioxidant system pathways under drought stress conditions (Santaniello et al., 2017), and SEs was able to mitigate the drought stress by regulating the expression of genes involved in ABA biosynthesis and ROS detoxification (Shukla et al., 2018).

Based on the above analysis, the effect of SEs on sugarcane yield was more obvious under drought conditions, which could be due to the fact that the SEs contained many active substances which were conducive to improving the drought tolerance of sugarcane (Spann and Little, 2011; Martynenko et al., 2016; Shukla et al., 2018). Therefore, it is suggested that SEs should be sprayed once both at seedling and early elongation stages in sugarcane management in rain-fed agricultural areas.

## Conclusion

In this study, spraying SEs on sugarcane leaves at seedling and early elongation stages promoted sugarcane growth in rainfed areas without irrigation. SE application promoted photosynthesis and transpiration, improved WUEI and utilization efficiency of nitrogen, phosphorus, and potassium, and increased the height of sugarcane, thus, increasing the yield and economic benefits. Moreover, in drought years, SEs had more significant effects on alleviating sugarcane yield decline caused by drought.

Furthermore, we found that spraying SEs at the early mature stage of sugarcane could regulate the activities of enzymes related to sugar accumulation in sugarcane leaves and stems, increase the activities of sucrose phosphate synthase in leaves and stems, and reduce the activities of soluble acid invertase in stems, which was conducive to promoting sucrose accumulation in sugarcane stems.

Due to the improvement effect of SEs on sugarcane yield and sucrose content in this research, we suggest that SEs should be sprayed at different growth stages in sugarcane production. It is better to spray three times at seedling, elongation, and early mature stages. These provide a theoretical basis for the application of SE in agricultural areas.

## Data Availability Statement

The original contributions presented in the study are included in the article/supplementary material, further inquiries can be directed to the corresponding author/s.

## Author Contributions

DC conceived and designed the experiments with the help of HS. DC, WZ, JA, DS, and YJ performed the field trials and agronomy study. DC analyzed the data and wrote many parts of the manuscript. YH performed the enzyme study and analyzed the data. WZ and JY performed and wrote the statistical analyses. DS, YH, and YJ performed sucrose and nutrient analysis. ZH prepared the figures. DC wrote the first draft of the manuscript with the help of JY, WZ, and HS. ZH and DC prepared the final manuscript with the help of HS. All authors contributed to the article and approved the submitted version.

## Conflict of Interest

The authors declare that the research was conducted in the absence of any commercial or financial relationships that could be construed as a potential conflict of interest.
